# Formulation Considerations for Autologous T Cell Drug Products

**DOI:** 10.3390/pharmaceutics13081317

**Published:** 2021-08-23

**Authors:** Christopher F. van der Walle, Sonya Godbert, Gabriele Saito, Zein Azhari

**Affiliations:** GSK R&D, Gunnels Wood, Stevenage, Herts SG1 2NY, UK; sonya.x.godbert@gsk.com (S.G.); gabrielesaito84@googlemail.com (G.S.); zein.x.azhari@gsk.com (Z.A.)

**Keywords:** closed process, fill–finish, design space, primary container, cryopreservation, freeze–thaw

## Abstract

Genetically modified autologous T cells have become an established immunotherapy in the fight against cancer. The manufacture of chimeric antigen receptor (CAR) and αβ-T cell receptor (TCR) transduced T cells poses unique challenges, including the formulation, cryopreservation and fill–finish steps, which are the focus of this review. With an increasing number of marketing approvals for CAR-T cell therapies, comparison of their formulation design and presentation for administration can be made. These differences will be discussed alongside the emergence of automated formulation and fill-finish processes, the formulation design space, Monte Carlo simulation applied to risk analysis, primary container selection, freezing profiles and thaw and the use of dimethyl sulfoxide and alternative solvents/excipients as cryopreservation agents. The review will conclude with a discussion of the pharmaceutical solutions required to meet the simplification of manufacture and flexibility in dosage form for clinical treatment.

## 1. Introduction

There is considerable excitement surrounding the treatment potential of autologous T cell immunotherapies. Rather than conventional cancer therapy, which seeks to eradicate cancerous cells using cytotoxic chemicals, radiation and/or surgery, immunotherapy seeks the same goal by stimulating the immune system. T cells are key players in adaptive, cell-mediated immunity and can be genetically engineered (‘programmed’) to target specific tumour cells. This can involve the design of a chimeric antigen receptor (CAR) or αβ-T cell receptor (TCR) transgene, coded into a vector such as a lentivirus for transduction of the T cell [1]. A publication in 2011 of a pilot clinical trial for patients receiving B cell antigen CD19-directed CAR-T cell therapy for chronic lymphocytic leukaemia was followed by a 2013 publication from the same group for paediatric patients receiving CTL019 CAR-T cell therapy for acute lymphoid leukaemia (ALL) [2,3]. The CTL019 transgene is a fusion of an anti-CD19 murine single-chain antibody fragment with CD137 (costimulatory domain enhancing cell proliferation) and CD3-ζ (transduction domain stimulating intracellular signalling upon CD19 antigen binding). In collaboration with Novartis, CTL019 became tisagenlecleucel (Kymriah^®^), the first cell-based gene therapy approved by the U.S. Food and Drug Administration (FDA) in 2017. To date, there are no approved TCR-T cell therapies, although their number in clinical trials increases year-on-year [4], as for CAR-T cell therapies [5].

In contrast to CAR-T cells, which bind native cell surface antigens, TCR-T cells access a wider range of intracellular peptide antigens presented by the major histocompatibility complex [1]. In both cases, to avoid graft versus host rejection upon their infusion for treatment of a cancer patient, the T cells must first be obtained from the same patient, i.e., autologous. The vein-to-vein manufacturing process and scale-up is outside the scope of this review (cf. [6]) but key steps include: apheresis to collect a patient’s peripheral blood mononuclear cells (PBMCs), T cell enrichment with anti-CD3/anti-CD28 antibodies, virus-mediated transduction and expansion ex vivo. A simplified process would be afforded by allogenic ‘off-the-shelf’ T cells, which are intended as ‘universal donors’ [7]. Nevertheless, aspects of their formulation, such as cryopreservation and fill–finish into bags, are common with autologous T cells. The formulator needs to consider the dose, i.e., the number of CD3+ transduced T cells as a fraction of the total number of nucleated cells infused in a particular volume of media provided at the patient’s bedside in a sufficient number of bags for intravenous (IV) infusion. These five attributes would be included amongst others in the Quality Target Product Profile (QTPP) referred to in the Pharmaceutical Development quality guideline Q8(R2) [8], which may evolve between Phase I and III clinical trials as the treatment regimen for the cancer becomes increasingly understood by the clinicians.

A review of the formulation of a particular drug modality may be expected to start with the excipient(s); however, cell formulation is necessarily distinct from other biologics in that the formulation must maintain cell metabolism and function (T cells are ‘living drugs’). This review therefore starts with the selected attributes referred to above—dose and volume—before moving on to single-use plasticware for cryogenic storage, freeze–thaw profiles and cryoprotectants, and concluding with future perspectives.

## 2. The T Cell Dose

Following infusion to the patient, CAR-T cells continue to proliferate in vivo and have been shown to persist in some patients for 4 years, correlating with therapeutic efficacy on account of sustained functionality [9]. The pharmacokinetic profiles of marketed CAR-T cell therapies generally show peak levels (T_max_) at 1–2 weeks and responders have significantly higher total drug exposure (AUC_0–28 d_) and maximum peak concentration (C_max_) [10,11,12,13,14]. Phase I clinical trial design for dose optimisation of CAR-T cells remains a topic of debate but typically involves a variation on the 3 + 3 dose escalation [15], i.e., based on the assumption that dose versus toxicity cannot be modelled other than by simple scaling and monitoring, three patients are given a dose considered safe, followed by two more three-patient cohorts receiving fixed dose increments. For example, ten patients with relapsed/refractory B-cell non-Hodgkin’s lymphoma in a Phase I study of anti-CD19 CAR-T cells received an escalating dose of 2.5 × 10^7^ (*n* = 3), 5 × 10^7^ (*n* = 4) and 1 × 10^8^ (*n* = 3) CAR-positive viable T cells [16]. The dose, primary container and fill volume for the five FDA-approved CAR-T cell therapies are shown in Table 1.

TCR-T cell therapies may require fill into several bags because the doses (in cell number) are at least one order of magnitude greater (Table 2). For example, in a recent clinical trial conducted at Shenzhen Second People’s Hospital, four human leukocyte antigen-A2-positive patients with non-small cell lung cancer received in the range of 1.67–10.60 × 10^9^ New York oesophageal squamous cell carcinoma-1 TCR-T cells (median 7.16 × 10^9^ cells, transduction efficiency 36.6–96.6%), intravenously infused over one to three days [17]. Upon a cursory comparison of the above-mentioned doses, fill volumes and primary containers, it is apparent that the formulator has relatively limited options for dosage form design to meet the QTPP, i.e., cryopreserved drug product in cryobags/vials for IV infusion with T cell concentrations over a range of 10^6^–10^8^ CAR/TCR T cells/mL.

## 3. Towards Automated Formulation

Before a manufacturing strategy can be considered, it should be remembered that its feasibility, built on processes using leukapheresis material from healthy donors, must translate to leukapheresis material from cancer patients. The quality and quantity of PBMCs harvested from cancer patients will be imprinted by the disease progression, immune function and exposure to chemotherapeutics causing lymphocytopenia (low lymphocyte blood count); their characteristics will in turn affect T cell transduction efficiency, proliferation, phenotype (effector/central memory, CD4+/CD8+ ratios, etc.) and ultimately the dose that can be manufactured [15]. The routine manufacture of autologous T cells therefore remains very challenging. Key manufacturing steps within the vein-to-vein time referred to above demand complex and lengthy workflows, measured in days to weeks. Streamlining the T cell manufacturing process is an important strategy for reducing the cost of goods (COGs) for T cell therapies [18]. The Chemistry, Manufacturing and Controls (CMC) strategy can aim to reduce vein-to-vein times but the aseptic filling of T cell dispersions into primary containers is inherently time-constrained on account of T cell drug product stability at around room temperature in the presence of the cryopreservation agent, typically dimethyl sulfoxide (DMSO). In combination, these factors, drive the formulation and fill–finish steps towards a closed process that is either fully or semi-automated and seamless with upstream cell processing steps.

Under Good Manufacturing Practice (GMP), a closed end-to-end workflow for sterile products that operates in an ISO 14644-1 Class 7 cleanroom (equal to FDA Class 10,000 and EU grade C) has clear advantages over an open process requiring intensive manual steps in an ISO 14644-1 Class 5 cleanroom (equivalent to FDA Class 100 and approximately equal to EU grade A). This has been demonstrated at the National Institutes of Health Clinical Center, supplying TCR-T cell therapies to Phase I/II clinical trials, wherein a modular system was introduced encompassing two phases [19]. The first phase consisted of closed lymphocyte enrichment, anti-CD3 activation in culture flasks and transfer to culture bags for transduction by retrovirus. A consistent transduction efficiency of 73–90% was observed, dependent on the TCR transgene, with 93% cell viability and >96% CD3+ T cells by day 7. The second phase consisted of the transfer of cells from the culture bag to a closed culture apparatus, retaining T cell transduction efficiency and functionality during an 1890-fold expansion by day 14 (final yield of 18.9 (±1.4) × 10^9^ T cells). More advanced culture apparatus has enabled fully closed, automated end-to-end processes; an example report demonstrated a yield of 2.9 (±0.7) × 10^9^ T cells after 10 day expansion (55% transduction efficiency with lentivirus harbouring a CAR transgene, 96.5% viability and 98% CD3+ T cells) [20]. Thus, T cell expansion to numbers sufficient for the above-mentioned CAR-T and TCR-T cell therapeutic doses are within the capabilities of either semi- or fully closed, automated culture apparatus.

Following the expansion step and counting of the total number of nucleated cells (TNC) in the bulk drug substance, the formulation step involves cell concentration/wash and resuspension/dilution into a final buffer containing DMSO to a target TNC concentration for the drug product [19]. In a manual process, the target TNC/mL value may simply be fixed, and the formulator makes necessary resuspension/dilution steps and fill into bag(s). Bags may be over-wrapped and placed into metal cassettes to ensure that their thickness is uniform during freezing. Metal is also an excellent heat conductor and facilitates uniform cooling on freezing and thawing across the cassette and bag [21]. In a closed, automated fill–finish process, the TNC/mL value for the drug substance calculates the cell concentration and dilution steps to meet a target TNC/mL for the drug product [20]. The drug product volume, minus the volume for quality control, is then split across ≥1 bag(s) with reference to the validated fill volume of the bag, e.g., 10–30 mL fill for a bag of 50 mL nominal volume. The transduction efficiency calculates the number of CAR/TCR positive T cells, a value that should meet the therapeutic dose and is listed on the drug product label. Despite the requirement for the T cell drug product to meet release specifications (Table 3), there have been instances where carefully managed patient access to out of specification CAR-T cell batches has been permitted [22].

## 4. Fill–Finish and Monte Carlo Simulation

A peristaltic pump generating flow in a supported section of silicone tubing that is integral to the single-use set (SUS) is a convenient solution to maintaining a closed end-to-end T cell process. With all tubing, filters, connectors and bags pre-sterilised, the closed fill–finish workflow can ensure product quality in an ISO 14644-1 Class 7 (minimum) cleanroom [24]. For low-viscosity cell dispersions, peristaltic pumps generating precise, pulsation-free flow in silicon tubing can fill drug product over large volume ranges into bags of differing size and number [25]. The possible entrainment of air from a bulk bag into a drug product bag(s) remains a concern and monitoring bag weight (ideally in-process, automated) may be considered [26]. Mixing efficiencies of buffer components requires attention since the excipient concentrations must remain within the formulation design space defined during the pharmaceutical development studies. Computational fluid dynamics could be used to relate mixing efficiency to wave bag/chamber dimensions, fill volumes and rocking angle/stirring speed, building on recent simulations for fluid flow in wave bags and stirred tank bioreactors [27,28]. In practice, confirmation of the target DMSO concentration may be inferred by proxy through comparison of osmolality and T cell quality/quantity before and after the formulation step and post-thaw. Analysing the risk of excipient concentrations lying outside the formulation design space can be computed by Monte Carlo simulation applied to failure mode effects analysis (FMEA) [29].

A hypothetical scenario is proposed wherein a T cell formulation involves the resuspension of the washed cell pellet in a 1:1 volume equivalent of two preformulated excipient solutions: 5% *w/v* human serum albumin (HSA) in saline, and CryoStor^®^ CS10 (10% *w*/*v* DMSO), as exemplified by Yescarta^®^ [14,30]. In this scenario, a ‘Design of Experiments’ approach (cf. [31]) could have been used to generate data showing that HSA concentrations must remain within 25% of a 2.5% *w*/*v* target, and DMSO concentrations must remain within 20% of a 5% *w*/*v* target. The accuracy of each dispensing step is given a 10% variance, based on a previous report for fill by peristaltic pump, albeit for smaller dispensed volumes [32]. The dispensing steps include: (i) T cell resuspension in a target volume of 5% HSA in saline, (ii) addition of CryoStor^®^ CS10 1:1 volume equivalent, (iii) mixing and fill into the primary container at the desired volume. To proceed with a statistical simulation of the formulation process, the following equations can be applied:

% *w*/*v* HSA = 5*x*/(*x* + *y*)

% *w*/*v* DMSO = 10*y*/(*x* + *y*)

where *x* = volume of HSA–saline, and *y* = volume of CryoStor CS10.

**Figure 1:**
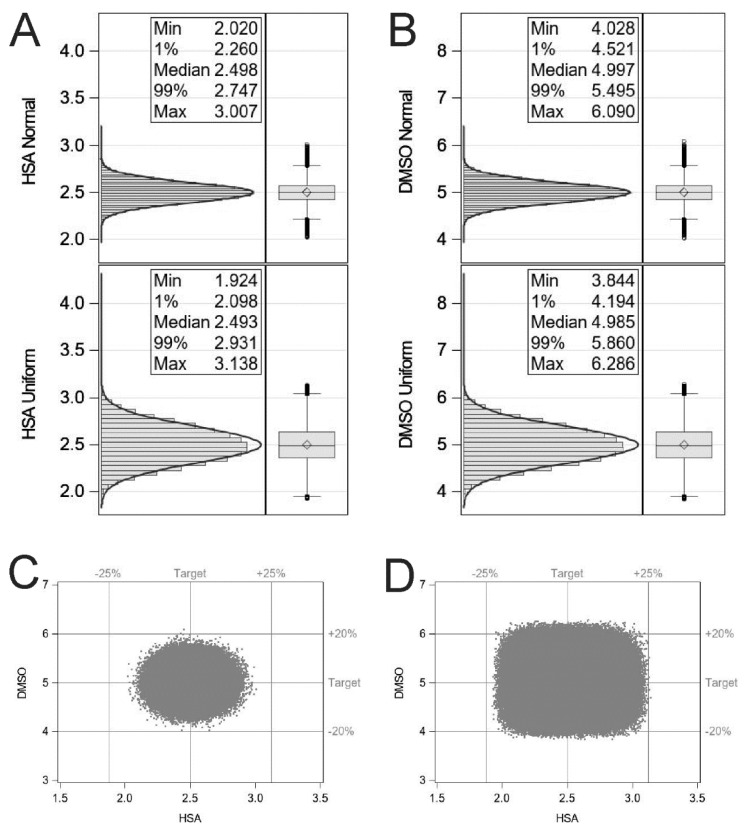
Histograms illustrating the effect of ±10% dispensing inaccuracy from either a normal distribution or uniform distribution for HSA (**A**) and DMSO (**B**). Scatter plots representing the Monte Carlo simulations for normal centred (**C**) and uniform (**D**) distributions. Statistical simulations were performed using the SAS System for Windows, release 9.4.

Simulating with the assumption that each dispensing step is independent and the formulation process is centred on the set point with a variance of 10%, the probability of obtaining a T cell formulation outside the design space was calculated as 154 parts per million. Whereas simulating with the assumption that each dispensing is equally likely to be anywhere within ±10% of the set point, the probability of obtaining a T cell formulation outside the design space was calculated as 278,231 parts per million (Figure 1).

## 5. Primary Container Selection

The primary container is that which is in immediate contact with the drug product and for currently marketed CAR-T products is a bag or vial able to withstand cryogenic storage. The current preference appears to be for ethylene-vinyl acetate (EVA) or polyolefin bags, of which there are several manufacturers. This represents a departure from the longer established use of polyvinyl chloride (PVC) as a material for cryogenic bags for blood products such as erythrocytes [33]. PVC is an amorphous thermoplastic and amenable to heat extrusion manufacture to generate sheets, tubes, etc. The glass transition temperature (T_g_) of amorphous and semi-crystalline polymers is a critical parameter that dictates its upper and lower functional temperature and helps to identify the parameters necessary to process the polymer. The molecular properties underpinning the glass transition of amorphous polymers are beyond the scope of this review. Superficially, above the T_g_, polymer chains in the amorphous phase can ‘slip’ and this imparts a ‘rubbery’ state, i.e., the plastic is soft, whereas below the T_g_, the polymer chains are restricted as regards their intermolecular mobility and this imparts a ‘glassy’ state, i.e., the plastic becomes hard. Knowledge of the T_g_ may help to determine adjustments for further polymer processing or synthesis to achieve the desired material properties for different applications (e.g., stiffness and flexibility at sub-ambient temperatures) [34]. The T_g_ of ‘pure’ PVC ranges from 350 to 360 K (*ca*. 52–62 °C), so plasticisers (additives that decrease the T_g_) are required to reduce the T_g_ to a value that permits freezing of blood products. A commonly used PVC plasticiser is di(2-ethylhexyl) phthalate (DEHP), which, at 25% weight percent (*w*/*w* %), reduces the T_g_ of PVC to around 0 °C; 30 to 40 *w*/*w* % DEHP reduces the T_g_ of PVC to around −10 to −30 °C, respectively [35]. DEHP has proved controversial since it leaches into the blood product and, although its presence stabilises the erythrocyte cell membrane (promoting stability during freezing), it is a known carcinogen and endocrine-disrupting agent to humans and the environment [33]. Extraction of the plasticiser over the long term also makes PVC less flexible [36]. Thus, PVC is unsuitable as the primary container material for T cells, though its use continues as a material for tubing for the filling assembly connected to EVA/polyolefin bags.

Alternative thermoplastics that can be similarly extruded at scale and sealed to form an aseptic weld in either sheet or tube form include semi-crystalline EVA and polyolefins such as polyethylene (PE) and cyclic olefin copolymer (COC). The T_g_ of linear, unbranched PE has been extensively studied but a consensus has proved elusive because of the difficulty in controlling the % crystallinity. Although T_g_ values ranging from −10 to −100 °C are reported, for entirely amorphous PE, a T_g_ of −23 °C can be obtained by extrapolation, irrespective of material density [37]. The T_g_ of EVA is dependent upon the % *w*/*w* of vinyl acetate monomer and the degree of crystallinity: T_g_ values between −20 and −30 °C have been measured for 8–46% crystalline EVA containing 9–40% *w*/*w* vinyl acetate, with corresponding melting temperatures (T_m_) of 45–102 °C [38]. Primary containers fabricated from EVA are therefore suitable for the freezing of T cell products but must be sterilised by gamma-irradiation rather than by steam under pressure (120 °C) as this would exceed the reported T_m_ of EVA. The cryogenic vials suitable for the storage of PBMCs are not made from borosilicate type I glass but rather polypropylene or, more recently, COC [39]. An example of COC vials that are documented for the cryogenic freezing and storage of PBMCs and T cells are CellSeal^®^ vials (Cook Regentec, Indianapolis, IN 46202, United States) [40]. Similar to EVA bags, the CellSeal^®^ vials have EVA tubing that enables manual fill (in an ISO 14644-1 Class 7 cleanroom) or automated fill in a closed system. The EVA tube is aseptically sealed using an appropriate sealing device and the container closure integrity of the vials through freezing, storage, shipping and thaw has been demonstrated using dye ingress and microbial challenge tests [41].

EVA is permeable to gaseous CO_2_ and O_2_, which is necessary to maintain cell metabolism and viability between the T cell fill–finish step and administration. There was no direct relationship observed between the % *w*/*w* VA and the permeability coefficient (*P*) of CO_2_ and O_2_ across EVA films with thicknesses ranging between ca. 100 and 250 μm. Instead, *P* scaled inversely with T_g_ and was an order of magnitude larger for CO_2_ versus O_2_ [42]. For water vapour, *P* scaled with the % *w*/*w* VA and was between 3 and 4 orders of magnitude larger than *P* for CO_2_ and O_2_, respectively. In contrast, PVC and PE have low permeability to water vapour, CO_2_ and O_2_ (Table 4) [43]. It was hypothesised that there is only a moderate affinity between the various gases and the polymers, while the permeability is determined by the relative effects of the % crystallinity and chain mobility in the amorphous phase. It is noteworthy that T cell primary containers are not composed of laminar composites of EVA with ethyl vinyl alcohol (EVOH), since the latter has exceptionally poor O_2_ permeability (and is used where oxidation of biologics during storage is considered a risk) [44].

### Failure of the Primary Container

The primary container must be sufficiently robust to withstand the cryogenic temperatures of shipping conditions followed by thawing to ambient temperature. As mentioned above, a metal cassette is required for controlled freezing but it also allows for efficient storage and shipping protection. Secondary packaging (overwrapping, where used) restricts the movement of the filled bag in the cassette, adds protection and prevents wetting of the primary container during thaw in water baths. Nevertheless, bag failure does occur, though it is not frequently reported in the literature. Mele et al. reported a 1.06% rupture rate (4 bags) for 377 reinfused bags of peripheral blood progenitor cells (PBPCs) formulated in 10% DMSO and filled in polyethylene/EVA bags from one supplier [45]. Of the four bags, three fractured during removal from the liquid nitrogen tank and one due to accidental damage. Thyagarajan et al. reported bag failure for cryopreserved umbilical cord blood for the years 2000–2006 for several bag types and suppliers. Of 679 drug products transplanted, 24 bags (3.5%) failed overall, with 17 bag failures detected upon thaw and the remainder detected upon receipt and inspection of the drug product [46]. All bag failures but one were considered minor on account of a break or tear at either the ports, tubing or bag seam, and most had been in cryogenic storage for >2 years. These minor bag failures did not lead to ‘significant compromise’ of the drug product or ‘adverse clinical outcome’. Khuu et al. recorded the EVA bag failure rates for reinfusion of PBPC or lymphocyte drug products formulated in 5% or 10% DMSO at volumes of 25–75 mL, overwrapped, cryopreserved and stored in liquid/vapour phase nitrogen [47]. The authors reported that the bag failure rates for three periods across 2000–2002 were 1.7% (10/599), 9.6% (58/605) and 0% (0/177). The high failure rate was attributed to specific bag lots (batches) from one supplier rather than product type, formulation, liquid versus vapour phase storage or freezer location.

## 6. Particulates, Extractables and Leachables

A review of process-related impurities derived from the production of viral vector critical starting material and safety testing of the T cell drug product for adventitious agents is beyond the scope of this review (cf. [48]). Here will be considered particulates, extractables and leachables derived from the single-use plasticware required for T cell formulation and fill into a primary container. With the adoption of a SUS for T cell processing, the potential contamination from process changeover and clean/steam-in-place is eliminated but the SUS materials must have better performance, purity and be practically free of particulates. Unless described in the relevant pharmacopoeia or approved for use in food packaging, regulatory guidance requires data detailing compatibility with the drug product ascertained by extraction and interaction studies, toxicology and the effect of sterilisation [49]. Extractables are chemicals that migrate out of the SUS under exaggerated conditions and encompass leachables that migrate out over the shelf-life of the drug product. The BioPhorum Operations Group uses a 70-day period under solvent, acid, base and water conditions to extract chemicals from the SUS prior to their quantitative analysis using hyphenated methods such as gas/liquid chromatography–mass spectrometry (Table 5) [50].

Particulates are similarly controlled by the regulatory agencies because, if infused, they have the potential to occlude blood vessels and damage tissue/organs dependent on their size, shape, composition and quantity. Although SUSs are produced in clean rooms (cf. ISO 14644-1), the possibility of particulates adhering to plastic sheeting and being subsequently washed off into the primary container during drug product fill cannot be ruled out. Indeed, parenterals must be ‘essentially free’ from visible particles [51]. A report using membrane microscopy measurement of partially water-filled 20 L SUS bags suggests that such incidences of particulates can occur [52]. The pharmacopoeia also describes tests for subvisible (10–100 μm) particulates [53] and, in one report, six different types of 1 L SUS bags were rinsed with filtered water and measured using both an in-line high-resolution camera and off-line USP <788> [54]. In-line measurements recorded consistently higher numbers than the off-line test. This demonstrates that a gap exists for SUS manufacturers adapting compendial particle tests intended only for drug products. Industry-standardised methods specifically related to the rinsing of particles from SUS surfaces with water or water plus salt(s) to generate a suspension for quantitative analysis have been proposed by the American Society for Testing and Materials [55]. It should be noted that the application of compendial methods for subvisible particulate testing to cell therapies is counter-intuitive on the basis that the cells are themselves a dispersion of subvisible particles. Nevertheless, using Dynabeads as potential subvisible particulate impurities arising from the T cell process, Grabarek et al. used flow imaging microscopy and image classification via a convolutional neural network to distinguish such impurities from immortalised T lymphocytes (Jurkat cells) [56].

## 7. Freeze and Thaw of T Cell Drug Product

A detailed review of ice formation and morphology in the context of cell cryopreservation is beyond the scope of this review (cf. [57]). Cell-membrane-permeable cryoprotectants facilitate intracellular vitrification upon freezing (i.e., amorphous ice formation, avoiding mechanical damage by intracellular ice crystals) and increase intracellular osmolality, which may mitigate cell dehydration (saturated solutes in supercooled water in the extracellular space generating high osmotic pressures). Levin outlined a theoretical protocol for maintaining a constant intracellular water volume by introducing permeable and non-permeable cryoprotectants to the extracellular solution such that osmotic forces are balanced and the transmembrane water flux remains zero [58]. While interesting, this protocol was for an isolated cell and its translation to T cell processing is required. Cell-permeable cryoprotectants also partition into the lipid membrane, the conformational order and density of which can be quantified by Fourier-transform infrared spectroscopy (FTIR) during freeze–thaw. For example, increased lipid packing was monitored for fibroblasts frozen in the absence of a cryoprotectant, whereas 10% glycerol or 10% DMSO preserved the cell membrane structure and post-thaw viability [59]. Interestingly, cell dehydration has been shown not to occur during isochoric (constant volume) freezing, although this novel technology remains untested in cell immunotherapy [60].

The rate of freezing is the means by which ice nucleation and growth can be controlled for a given DMSO concentration. Briefly, a liquid nitrogen (LN) or LN-free controlled-rate freezer (CRF, the equipment of which is reviewed elsewhere [61]) is commonly used during cell drug product development studies to determine an appropriate freeze cycle, prior to long-term storage in vapour-phase LN (−150 °C) or LN (−196 °C). Passive freezing methods typically involve placing cell sample tubes in isopropyl alcohol in a −80 °C freezer and can attenuate spontaneous ice nucleation, improving the recovery of culture-enriched T cells [62]. Alcohol-free passive freezing devices have been demonstrated within the Ovasave^®^ Phase 2b clinical trial to be scalable, reproducible and suitable for GMP manufacturing environments [63].

**Figure 2:**
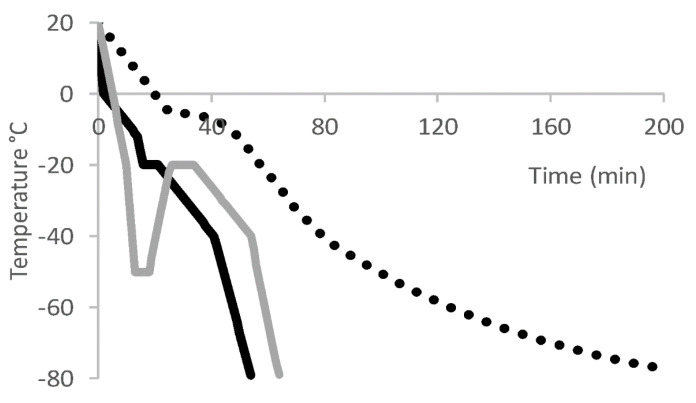
Example freezing rate profiles used to cryopreserve T cell. Dotted black line = passive freezing [63]; solid black line = CRF with 5-min hold time at −20 °C (dissipate heat of crystallisation) [65]; solid gray line = CRF with annealing step from −50 to −20 °C followed by a 10-min hold.

However, a CRF is considered to offer advantages over passive freezing because multistep profiles are possible and freezing rates can be controlled to −1 °C/min or less [39]. For example, Great Ormond Street Hospital describes the preferential use of a CRF for formulated leukapheresis material from patients (by addition of 1:4 DMSO:HSA 4.5% solution) prior to shipping to Novartis for CAR-T cell Kymriah^®^ manufacture [64]. A direct comparison of passive freezing versus a CRF for leukapheresis material from healthy donors concluded that only CRF enabled the full maturation of (dendritic) cells with the relevant surface markers, cytokine production and function [65]. A CRF can also enable the programming of an annealing (thermal treatment) step in which a frozen product is temporarily warmed and re-cooled, e.g., −50 °C to −20 °C for 2 h, then back to −50° C (Figure 2). If a rapid cool step (≥ −10 °C/min) is first used, resulting in incomplete ice crystallisation (i.e., a fraction is metastable amorphous ice), then annealing will progress to the formation of ice crystals. This avoids potential devitrification upon thaw (discussed below) and may also attenuate fracturing and damage to the product. In the manufacture of CD-19-specific T cells for the clinic, following formulation in HSA (2% *v*/*v*): Plasmalyte: DMSO (5:4:1) and fill into EVA bags, a CRF was used to achieve an annealing step following a rapid cool step, with long-term storage in vapour-phase LN [66].

Unfortunately, a consensus on the preferred freezing profiles for T cell drug products has not been reached and de-risking temperature excursions during shipping or storage requires case-by-case assessment. However, the Fraunhofer Institut für Biomedizinische Technik (IBMT) developed a novel robotic system enabling accurate temperature cycling of frozen PBMCs at a concentration of 10^7^ cell/mL, formulated in ‘IBMT-media I’ (10% DMSO and 1% Poloxamer 188 [67]) [68]. Two temperature cycles relating directly to the sample temperature were used: (i) cycling 400 × between −135 °C and −60 °C, (ii) cycling 400 × between −135 °C and −102 °C. For samples thawed at 37 °C and assayed, only cycling to −60 °C caused a significant loss in PBMC viability and antigen-specific T cell response (ca. −3% and −30%, respectively) compared to samples held at −135 °C constantly.

T cells are infused immediately after complete thaw of the infusion bag ‘at the patient’s bedside’ without further dilution. This raises the question of how best to proceed with the thaw step, since there exist several options. In theory, slow thaw should be avoided to prevent de-vitrification, i.e., the formation of ice crystals within amorphous ice when warmed below the critical warming rate [69]. Uniformity of heating must be ensured to prevent thermal strain resulting in physical stress [70]. In practice, thaw devices used in clinics include water baths maintained at 37 °C as well as more sophisticated devices with water-warmed cushions or dry-block devices, which de-risk water-borne contaminants. Electronic temperature recording and tracking systems are available for the latter devices and can support the required Chain-of-Custody strategy (cf. [21]). Thawing of typical T cell drug product volumes (Table 1) using a water bath is generally complete in approx. 5 min. Baboo et al. showed that T cells thawed in a water bath after slow freezing (−1 °C/min) retain near 100% viability but after rapid freezing (−10 °C/min) retain only ~80% viability upon slow thawing in air [39]. Corresponding cryomicroscopy images showed that a ‘very slow thaw’ (−80 to 0 °C in approx. 1 h) following rapid freezing induced ice recrystallisation in CryoStor^®^ CS10 (10% DMSO). It would be interesting to expand this work by modelling and experimentally defining the critical warming rates for different formulation buffers encompassing a range of percentages of DMSO and albumin.

## 8. The Physical Effects of DMSO on Cells

Having established that DMSO is a highly effective cell cryoprotectant and yet has cytotoxic effects, a molecular understanding of its action on lipid membranes and intracellular macromolecules is required. Molecular dynamic simulations of the interaction of DMSO molecules with suspended dipalmitoyl-phosphatidylcholine show that DMSO inserts below the lipid head-groups to disrupt lipid–lipid alignment, decreasing the rigidity of the bilayer and promoting membrane fusion. Structural deformation to organelles and the cell membrane during freeze and/or thaw can thus be accommodated more readily, a key mechanism for the cryoprotective effect of DMSO [71]. The same study and another [72], using a dioleoyl-phosphatidylcholine/20% cholesterol membrane system, showed that high DMSO concentrations of >20 mol% form ‘water pores’ in the bilayer. Pore formation was attributed to DMSO molecules transiently stabilising a ‘water structure’ between the lipid leaflets, with the lipid head groups reorienting to stabilise the water column, forming a pore.

The hypothesis that DMSO induces destabilisation of multimeric proteins (i.e., shifts the monomer ↔ multimer equilibrium to the left) is difficult to test in an appropriate intracellular context. It is well established that in vitro DMSO enables the preferential solvation of partially folded states [73] and disorders the α-helical structure at high (molar) concentrations [74] but at low (mM) concentrations stabilises electrostatic interactions between polar amino acid side chains and therefore β-sheet structure as a consequence of the decrease in solvent dielectric constant [75]. Nevertheless, intracellular studies show that 5–10% DMSO rapidly augments actin microfilament formation in a reversible process [76,77], with an overall shift in intracellular protein structure favouring β-sheet over α-helix for cells incubated in 0.1–1.5% DMSO [78], consistent with in vitro data [75].

DMSO exerts a high osmolality on the T cell formulation: even a low concentration of 5% *v*/*v* DMSO equates to 704 mOsmol/kg, calculated theoretically from the molar concentration and density of 1.1 g/cm^3^. This is significantly higher than that of blood, which ranges between 285 and 310 mOsmol/kg. Therefore, the measurement of the osmolality of T cell formulations should come within the wider analytical characterisation of the drug product [79]. Cell responses to high osmolarity are complex on account of actions both on the cell membrane and actin cytoskeleton. For example, in (Ap3 knockdown) T cells, the consequent disruption of cortical actomyosin invokes cell membrane detachment and ‘blebbing’, which, in this case, is reversible and associated with motility, rather than an indication of apoptosis [80]. HeLa cells exposed to 10% DMSO or 10% glycerol for 30 min developed 15–25% of the maximal number of blebs observed (at 20% DMSO or 30% glycerol, above which cell mortality rapidly increased from zero) [81]. Fibroblasts slightly swelled when exposed to 4300 mOsmol/kg sorbitol solution for 10 min and developed large blebs when returned to isotonic solution, accompanied by ~60% loss of filamentous actin [59]. When these experiments were repeated with PEG600, the fibroblasts shrunk in the hyperosmolar solution and developed only small blebs with no loss of F-actin when returned to the isotonic solution [82]. The authors suggested that the difference was due to the cell uptake of sorbitol but not PEG600. Translation of these data to aid the interpretation of T cell viability following suspension in different DMSO concentrations merely exposes our lack of knowledge of T cell responses to hyperosmolar solutions.

## 9. Potential Formulation Strategies for Reducing DMSO in Drug Product

The in vivo toxicity of DMSO is well documented [83], so the formulator must consider the absolute amount of DMSO in the drug product volume to be infused, especially to children. The European Medicines Agency guidance from the Committee for Advanced Therapies (CAT) is that ‘the plasma volume of the child needs to be calculated and the DMSO concentration after administration needs to be less than 1% of this volume’ [84], based on a review by Cox et al. [85]. Estimation of plasma volumes in children can be made with reference to data describing the variation in blood volume in prepubertal, pubertal and postpubertal stages in girls and boys by body mass, surface area or height [86]. In practice, for small infusion volumes between 10 and 50 mL, exemplified by Kymriah^®^, the corresponding volume of DMSO infused will be well below the CAT limit of 1% plasma volume.

Before consideration of alternative (novel) excipients to DMSO, options for its complete removal post-thaw or reduction in concentration in the cell drug product should be considered. Achieving the former would logically require a post-thaw cell processing step immediately prior to administration at the clinical site, such as cell washing by centrifugation and resuspension or step-wise dilution in DMSO-free media. In the case of hematopoietic stem cell transplantation, because of the relatively large volumes infused (hundreds of millilitres), cell washing prior to infusion has received considerable attention and commercial devices are available [87]. However, this scenario is not available to T cell therapies, where infusion is started as soon as practically possible after thaw and the in-use shelf-life is limited from 30 min to 3 h [11,12]. More realistically, for T cell therapies, a reduction in the fraction volume of DMSO in the cell drug product should be sought; concentrations for marketed products are shown in Table 6.

A high-throughput excipient screening campaign for T cell formulation design is complicated by the need for culture conditions and complex, time-consuming cell-based assays. While medium-throughput formulation screening for T cell viability can be implemented using 96-well plates, 384-well plates may require consideration of gaseous exchange and evaporation. Moreover, 96-well plate-based image cytometry has been demonstrated to measure CAR-T cell proliferation, transduction efficiency and target cell killing, with similar accuracy to conventional methods, with the advantage of added time-course data [88]. Data sets pertaining to prospective formulations acquired for cells suspended in multi-well plates will require translation to cell processes and scales required for patient supply. A consensus regarding excipients and their concentration ranges will likely be achieved as more T cell drug products and associated patents describing the formulation space (e.g., [89]) become registered.

An albumin-free, chemically defined T cell formulation has been a target for some time. Early work with PBMCs showed that cell viability 24 h post-thaw was ca. 91% and 94% for protein-free media containing 5% and 10% DMSO, respectively [67]. Increasing the volume fraction of Multiple Electrolytes Injection, Type 1 (Plasma-Lyte A, Baxter Int. Inc.) to 50% *v*/*v* appears to facilitate the removal of HSA (Table 6). Greater assurance that the HSA sourced is free of adventitious agents such as viruses and transmissible spongiform encephalopathy (TSE) could be achieved by the use of recombinant human albumin, which favourably preserved the stability of mesenchymal stromal cells with 10% DMSO [90]. The use of 5% *w*/*v* Dextran 40 (a polysaccharide from glucose, *M*_r_ ≈ 40,000) for the cryopreservation of placental- and umbilical-cord blood [91] is also found in the formulation of Kymriah^®^ as 10% Dextran 40 (LMD)/5% Dextrose (255 mOsmol/kg) (Table 6). Although Health Canada issued a safety alert regarding the presence of crystals in 10% Dextran 40 (LMD)/5% Dextrose [92], this risk is mitigated by 10-fold dilution in the Kymriah^®^ formulation. While excipients clearly contribute to the COGs, a model for autologous CAR-T cell therapies suggests that only 18% of COGs derive from ‘materials’ and these are mainly associated with patient apheresis, disposables and lentivirus [18].

## 10. Novel Excipients and Formulations of T Cells

Concerns surrounding the use of DMSO, from safety limitations in children to chemical incompatibility during manufacture, continue to stimulate the search for alternative cryopreservation agents. Much of this search has focused on the cryopreservation of embryonic stem cells, hematopoietic cell lines, umbilical cord- and bone marrow-derived mesenchymal stem cells [93]. Examples include polyampholytes such as those based on methacrylates or poly-lysine [94] and pore-forming amphipathic pH-responsive polymers facilitating the intracellular entry of non-reducing cryoprotectant sugars [95], such as comb-like pseudopeptides harbouring alkyl side chains that mimic fusogenic proteins [96]. Despite the burgeoning knowledge of these novel polymers, their introduction as novel excipients in the clinic needs careful consideration of regulatory guidance documents.

Within a new drug application, novel excipients are described in the drug product section of the Common Technical Document (CTD) (3.2.P.4.6) as follows: ‘For excipient(s) used for the first time in a drug product or by a new route of administration, full details of manufacture, characterisation, and controls, with cross references to supporting safety data (nonclinical and/or clinical) should be provided according to the drug substance format’ [97]. Novelty therefore includes new combinations of excipients and/or excipient(s) used at a concentration above that referred to in the FDA Inactive Ingredient Database or the Handbook of Pharmaceutical Excipients [98,99]. Outside a new drug application, the pathway for the regulatory approval of novel excipients is not clear. This is considered by the International Pharmaceutical Excipients Council (IPEC) to be stifling innovation in drug formulation, particularly solid dosage forms for small-molecule drugs [100]. With T cells being a recent clinical phenomenon, registration of associated novel excipients will likely first be observed within drug filings. The FDA guidance for industry, ‘Nonclinical Studies for the Safety Evaluation of Pharmaceutical Excipients’, is aging [101], as is the EMA guidance for ‘Excipients in the Dossier for Application for Marketing Authorisation of a Medicinal Product’ [102]. To encourage innovation and use in novel excipients, the IPEC outlined plans in 2009 for a Novel Excipient Safety Evaluation Procedure to ‘evaluate compliance of excipient data with the FDA guidance on safety evaluation and to make recommendations to the excipient manufacturer if data gaps are noted in the excipient dossier’ via an independent committee [100]. In 2019, the FDA requested information and comments (closure date February 2020) for a Novel Excipient Review Program Proposal to ‘reassure drug developers that the novel excipient can be used in a drug development program while minimising the risk that safety concerns would be raised by FDA during application review’ [103]. In principle, this would de-risk the additional investment and development time (for the excipient) against the competitive advantages such as increased drug product stability, tolerability, device compatibility, shipping temperature, etc. In the U.S., an industry may protect its intellectual property surrounding a novel excipient by submitting a Type IV (Excipient) Drug Master File to the FDA that describes relevant CMC information. In the EU, the sponsor may use a Certification of Suitability of European Pharmacopoeia monograph to ensure confidentiality of data relating to a novel excipient in the CTD.

**Figure 3:**
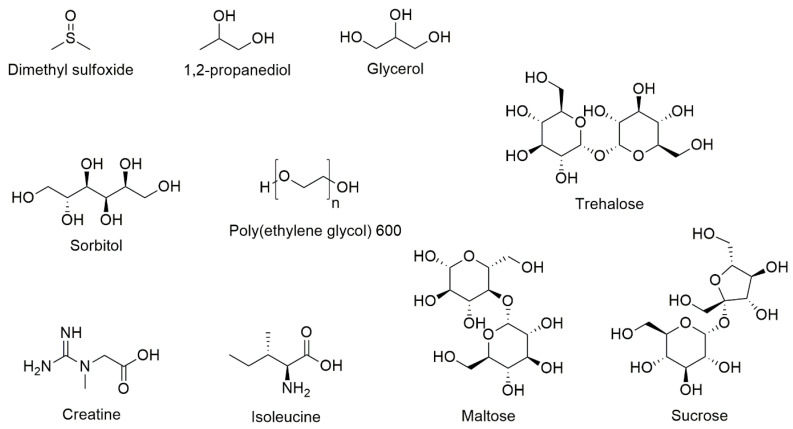
The chemical structures of excipients used in DMSO-free cryopreservation formulations of T cells.

Combinations of excipients, some used individually in marketed medicines, have been studied by the group of Hubel specifically in relation to T cell cryopreservation and would be considered novel from a regulatory viewpoint, as discussed above (Figure 3 and Table 7). Their study with Jurkat cells showed that it is possible to maintain post-thaw recoveries of 83 ± 5% in trehalose:glycerol:creatine (61 mM:10%:7 mM) and 79 ± 7% in sucrose:glycerol:creatine (438 mM:10%:10 mM), compared to 85 ± 5% for DMSO (10%) [104]. Trending the data across the six levels of excipient concentration studied, a non-linear increase in post-thaw recovery was observed for increasing glycerol concentrations (to 10%), whereas a broad peak was observed for the non-reducing disaccharides that was dependent on the glycerol (but not creatine) concentration.

A parallel study focused on sucrose:glycerol:isoleucine compositions defined the optimal formulation to be 146 mM:10%:43 mM, as measured by post-thaw recovery (max. 84%) of Jurkat cells [106]. Given the current use of hyperosmolar 5–10% DMSO solutions for T cell cryopreservation, it is noteworthy that both studies show no significant correlation between osmolarity (from ca. 200 to 1600 mOsm/kg) and the post-thaw recovery of the Jurkat cells. A recent paper from the same group evinced that sucrose could be substituted for trehalose at the same concentration and ratio to glycerol:isoleucine to maintain the post-thaw recovery of Jurkat cells and PBMCs near equivalent to 10% DMSO [107]. Substitution of sucrose for maltose was equally effective, although maltose is an interesting choice of disaccharide since it is a reducing sugar and risks the degradation of T cell surface proteins through the Maillard reaction, resulting in the formation of covalent adducts [108]. The group achieved a screen of excipient combinations using 96-well plates, each well (50 μL) containing 300,000 cells, with the plate frozen rapidly followed by an annealing step (−45 to −12 °C at +15 °C/min, to progress extracellular ice nucleation) before slow freezing to −60 °C, at −1 °C/min. Translation of these microscale experiments to scales required for patient supply, using appropriate T cell manufacturing processes (e.g., wave bags or closed-systems), would be interesting.

There is a surprisingly long list of commercially available DMSO-free media for cell cryopreservation and, while the majority are serum- or xeno-free, or chemically defined, few are ‘GMP grade’, and there is little or no evidence for their application to T cell lines, either primary or immortalised [109]. One exception includes Stem-CellBanker^®^ (Zenoaq Resource Co., Ltd., Chiyoda-Ku, Tokyo 101-0062, Japan), which substitutes propylene glycol (10%) for DMSO, which has been tested with the Jurkat cell line and has an accepted FDA Master File. Substitution of propylene glycol or glycerol for DMSO does not alleviate concerns regarding hyperosmolality or in vivo toxicity. Glycerol is used as a cryopreservation agent at 40% concentration for erythrocytes and its removal to levels <1% is required before transfusion in order to prevent intravascular haemolysis [110]. The EMA have issued a document on the use of propylene glycol as an excipient for drugs administered intravenously, including tolerance and recommended limits in children and adults [111]. The EMA safety limit for 5 years up to 17 years and adults is 500 mg/kg propylene glycol per day, which lies in the range for DMSO levels in children. For adults, it would appear that the propylene glycol limit is more restrictive that than for DMSO, but the EMA document acknowledges that ‘higher doses may be administered, when justified on a case by case basis’.

## 11. Future Perspectives

Moving the formulation and fill–finish steps from manual, open processes to closed, automated processes will reduce manufacturing complexity, COGs, risk of contamination and cell exposure to DMSO prior to freezing. As industrial experience and knowledge of process parameters increases from vein-to-vein, more complex QTPPs can be achieved. From the viewpoint of the formulator, these will include a wider range of cell doses and concentrations, primary container presentation and co-formulation of T cell phenotypes. To support these targets, it will be necessary to advance research in cryopreservation so that osmolality, cell toxicity and exposure time to non-aqueous solvents can be reduced alongside a greater tolerance of different freezing rates. Advances in the performance of plastics will improve the robustness of the container closure at ultra-low temperatures and new welding/sealing processes will increase flexibility in SUS design and assembly. The FDA recently stated that ‘by 2025, we predict that the FDA will be approving 10 to 20 cell and gene therapy products a year’ and will ‘make maximum use of our expedited programs including regenerative medicine advanced therapy (RMAT) designation and accelerated approval’ [112]. Based on current approvals, autologous T cell immunotherapies will be a major proportion of these cell and gene therapy products and formulators have a key role to play in ensuring that they reach the patient.

## Figures and Tables

**Table 1:** Comparison of dose and fill across marketed T cell drug products [10,11,12,13,14].

T Cell Drug Product (Proprietary Name)	Dose, Adults (CAR-Positive Viable T Cells)	Primary Container, Number Supplied per Dose	Fill Volume Per Bag/Vial, Number Bags/Vials Supplied per Dose
brexucabtagene autoleucel (Tecartus™)	2 × 10^6^ per kg body weight, max. 2 × 10^8^	cryogenic infusion bag	~68 mL, one
axicabtagene ciloleucel (Yescarta^®^)	2 × 10^6^ per kg body weight, max. 2 × 10^8^	cryogenic infusion bag	~68 mL, one
Tisagenlecleucel (Kymriah^®^)	0.6–6.0 × 10^8^	cryogenic infusion bag	10–50 mL, one to three
lisocabtagene maraleucel (Breyanzi^®^)	0.5–1.1 × 10^8^ (CD8 and CD4 components, 1:1)	cryogenic vials	4.6 mL, one to four (for each CD8 and CD4 component)
idecabtagene vicleucel (Abecma^®^)	3.0–4.6 × 10^8^	cryogenic infusion bag, 50/250/500 mL nominal volume	within validated range, one or more

**Table 2:** CAR and TCR-T cell doses for blood and solid tumour indications, respectively ^1^.

ClinicalTrials.gov Identifier	Phase	Study Complete	Sponsor	Transgene (CAR/TCR)	Indication	Dose (CAR/TCR-T Cells)
NCT03289455	I/II	May 2020	Autolus Limited	CD19/22 CAR	B Cell ALL	1 to 5.0 × 10^6^/kg
NCT02030847	II	Apr 2018	University of Pennsylvania	CD19 CAR	B Cell ALL	1–5 × 10^8^ CART-19 cells administered via split dosing: 10% day 1 (1–5 × 10^7^), 30% day 2 (3 × 10^7^–1.5 × 10^8^), 60% day 3 (6 × 10^7^–3 × 10^8^)
NCT01626495	I/IIa	Jul 2019	University of Pennsylvania	CD19 CAR	R/R ^2^ Leukaemia/Lymphoma	10% day 0, 30% day 1, possibly 60% day 14+; total dose goal ~1.5 × 10^7^–5 × 10^9^ (~0.3 × 10^6^–1.0 × 10^8^/kg)
NCT02030834	IIa	Sep 2020	University of Pennsylvania	humanised CD19 CAR	Diffuse Large B Cell Lymphoma	total dose of 1–5 × 10^8^
NCT03232619	I/II	Sep 2020	Bioray Laboratories, Second Xiangya Hospital of Central South University	humanised CD19 CAR	B Cell ALL	0.5–5 × 10^6^/kg
NCT02550535/ NCT01621724	I/II	May 2018	Cell Medica Ltd., University College London, Cell Therapy Catapult	Wilms tumour antigen 1 TCR	Myelodys-plastic syndrome, acute myeloid leukaemia	≤2 × 10^7^/kg (Cohort 1) ≤1 × 10^8^/kg (Cohort 2)
NCT01567891	I/IIa	Jun 2017	Adaptimmune	NY-ESO-1 TCR	R/R Ovarian Cancer	1–6 × 10^9^
NCT00509288	II	Jul 2012	National Cancer Institute	HLA-A 0201 TCR	Metastatic Melanoma	5 × 10^8^ to 3 × 10^11^

^1^, Clinical trials referred to in this table were identified by searching ClinicalTrials.Gov (accessed on 15 March 2021), for Phase 2 studies of CAR and TCR-T cell therapies that had been completed, whose eligibility criteria included adults of 18–64 years old. ^2^, Refractory/Relapsed.

**Table 3:** Example specifications for T cell drug product.

Parameter	Specifications ^1^	Method ^2^
Identity	CAR/TCR presence	qPCR ^3^
Appearance	Colour	Ph. Eur. 2.2.2 (USP <631>)
Dose	Number CAR/TCR positive cells	Calculated (from cell viability and transduction efficiency)
Potency	T cell function Transduction Efficiency	Cell-based assay Flow Cytometry (% T cells expressing transgene)
Purity	Total cell count CD3+ cell count Cell viability Residual beads ^4^	Cell count Flow Cytometry (% CD3+ cells) qPCR Microscopy
Safety	Copies of transgene RCL ^5^ Sterility Mycoplasma Endotoxin	qPCR qPCR/cell-based assay Ph. Eur. 2.6.27 (USP <71>) Ph. Eur. 2.6.7 (USP <63>) Ph. Eur. 2.6.14 (USP <85>)

^1^, note that not all specifications listed here may be performed for drug product release, and their categorisation against the parameters listed may differ between drug products (cf. redacted Yescarta^®^ Biologics License Application [23]); ^2^, acceptance criteria are dependent on the drug substance/product (unless pharmacopeial) and therefore not shown; ^3^, quantitative (real-time) polymerase chain reaction; ^4^, when activation and expansion of T cells is stimulated by anti-CD3 and anti-CD28 antibodies covalently coupled to supramagnetic beads several microns in size; ^5^, Replication Competent Lentivirus (capable of infecting non-target cells).

**Table 4:** Comparison of gas permeability coefficients at 25 °C for EVA, PVC and PE.

Polymer/Property	EVA_19_	EVA_50_	EVA_70_	PVC	LDPE ^1^
% vinyl acetate	19	50	70	-	-
% crystallinity	29	6	0	0	0
*P* H_2_O_(g)_ (barrer) ^2^	1134	9357	12,287	239	73
*P* O_2_ (barrer)	5.3	8.1	3.6	0.08	2.3
*P* CO_2_ (barrer)	57	70	30	0.28	16
^3^ αH_2_O/O_2_	214	1155	3413	3034	32
αH_2_O/CO_2_	20	134	410	856	4.6
αCO_2_/O_2_	10.8	8.6	8.3	3.5	7.0

^1^, values for low-density polyethylene (LDPE) from [43], all other values from [42]; ^2^, one barrer = 10^−10^ (cm^3^_STP_ cm)/(cm^2^ s cmHg), where cm^3^_STP_ = standard cubic centimetre; ^3^, α is the ideal selectivity coefficient (ratio of *P* for two gases).

**Table 5:** Selected conditions for testing of extractables from SUS ^1^.

Component	Solvent				Time, at 40 °C		
	50% Ethanol	0.5 N NaOH	0.1 M Phosphoric Acid	WFI ^2^	24 h	21 days	70 days
Bags for long-term storage	X	X	X	X	X	X	X
Storage bag tubing	X	X	X	X	X	X	X
Storage bag ports	X	X	X	X	X	X	X
Bag ports	X	X	X	X	X	X	
Tubing	X	X	X	X	X	X	
Tubing connectors, overmolded junctions	X	X	X	X	X		
Aseptic connectors	X	X	X	X	X		
Sterilising/process filters	X	X	X	X	X		
O-rings, gaskets	X				X		

^1^, adapted from Biophorum Operations Group; ^2^, water for injections; ’X indicates that selected components are tested against each condition.

**Table 6:** Excipients used in marketed T cell formulations [10,11,12,13,14,30].

T Cell Drug Product (Proprietary Name)	HSA Conc. (%)	DMSO Conc. (%)	Other Excipients
brexucabtagene autoleucel (Tecartus™)	-	5	sodium chloride, injection
axicabtagene ciloleucel (Yescarta^®^)	2.5	5	sodium chloride, injection
Tisagenlecleucel (Kymriah^®^)	5.0	7.5	31.25% Plasma-Lyte A, 31.25% of 5% Dextrose/0.45% sodium chloride, 10% Dextran 40 (LMD)/5% Dextrose
lisocabtagene maraleucel (Breyanzi^®^)	0.25	7.5	24% (*v*/*v*) Multiple Electrolytes for Injection, Type 1
idecabtagene vicleucel (Abecma^®^)	0.0	5	50% Plasma-Lyte A

-, not disclosed.

**Table 7:** Basic chemical properties of excipients used in DMSO-free cryopreservation formulations of T cells.

Material	MW (g/mol)	Density (g/mL) (20 °C)	Viscosity (20 °C) (mPa·s)	LogP	Polar Surface Area (Å^2^)	Solubility in H_2_O (mg/mL) (20 °C)
DMSO	78.13	1.10	1.996	−1.35	17.07	miscible
1,2-propanediol	76.09	1.036	42	−0.92	40.5	miscible
Glycerol	92.09	1.261	1412	−1.76	60.7	miscible
Sorbitol	182.17	1.489	-	−2.2	121.38	2350
Poly(ethylene glycol) 600 ^1^	600	1.12	150–190	-	-	miscible
Creatine	131.13	1.33	-	−0.2	90.4	13.3 (18 °C)
Isoleucine	131.17	-	-	−1.7	63.32	34.4
Trehalose	342.3	1.58 (24 °C)	-	−3 ^2^	190	592
Maltose	342.3	1.54	-	−3 ^2^	189.53	780
Sucrose	342.3	1.59	-	−3.7	189.53	2120 (25 °C)

^1^, values from [105], all other values collated from pubchem.ncbi.nlm.nih.gov, drugbank.com and chemspider.com (accessed on 15 July 2021); ^2^, predicted.

## Data Availability

Not applicable.
